# Extracellular membrane vesicles from *Limosilactobacillus reuteri* strengthen the intestinal epithelial integrity, modulate cytokine responses and antagonize activation of TRPV1

**DOI:** 10.3389/fmicb.2022.1032202

**Published:** 2022-11-17

**Authors:** Yanhong Pang, Ludwig Ermann Lundberg, Manuel Mata Forsberg, David Ahl, Helena Bysell, Anton Pallin, Eva Sverremark-Ekström, Roger Karlsson, Hans Jonsson, Stefan Roos

**Affiliations:** ^1^Department of Molecular Sciences, Uppsala BioCenter, Swedish University of Agricultural Sciences, Uppsala, Sweden; ^2^BioGaia AB, Stockholm, Sweden; ^3^The Department of Molecular Biosciences, The Wenner-Gren Institute, Stockholm University, Stockholm, Sweden; ^4^Department of Medical Cell Biology, Uppsala University, Uppsala, Sweden; ^5^Department of Clinical Microbiology, Sahlgrenska University Hospital, Gothenburg, Sweden; ^6^Nanoxis Consulting AB, Gothenburg, Sweden

**Keywords:** extracellular membrane vesicles, *Limosilactobacillus reuteri*, microbe-host interaction, immune response, epithelial cells integrity, TRPV1 pain receptor, proteomics, probiotics

## Abstract

Bacterial extracellular membrane vesicles (MV) are potent mediators of microbe-host signals, and they are not only important in host-pathogen interactions but also for the interactions between mutualistic bacteria and their hosts. Studies of MV derived from probiotics could enhance the understanding of these universal signal entities, and here we have studied MV derived from *Limosilactobacillus reuteri* DSM 17938 and BG-R46. The production of MV increased with cultivation time and after oxygen stress. Mass spectrometry-based proteomics analyses revealed that the MV carried a large number of bacterial cell surface proteins, several predicted to be involved in host-bacteria interactions. A 5′-nucleotidase, which catalyze the conversion of AMP into the signal molecule adenosine, was one of these and analysis of enzymatic activity showed that *L. reuteri* BG-R46 derived MV exhibited the highest activity. We also detected the TLR2 activator lipoteichoic acid on the MV. In models for host interactions, we first observed that *L. reuteri* MV were internalized by Caco-2/HT29-MTX epithelial cells, and in a dose-dependent manner decreased the leakage caused by enterotoxigenic *Escherichia coli* by up to 65%. Furthermore, the MV upregulated IL-1β and IL-6 from peripheral blood mononuclear cells (PBMC), but also dampened IFN-γ and TNF-α responses in PBMC challenged with *Staphylococcus aureus*. Finally, we showed that MV from the *L. reuteri* strains have an antagonistic effect on the pain receptor transient receptor potential vanilloid 1 in a model with primary dorsal root ganglion cells from rats. In summary, we have shown that these mobile nanometer scale MV reproduce several biological effects of *L. reuteri* cells and that the production parameters and selection of strain have an impact on the activity of the MV. This could potentially provide key information for development of innovative and more efficient probiotic products.

## Introduction

In recent years, there has been an increased interest in extracellular membrane vesicles (MV), which are abundant in nature and released in an evolutionally conserved manner by different types of organisms (Raposo and Stoorvogel, 2013; van Niel et al., 2018). Additionally, synthetic lipid nanoparticles with similarities to MV (Skotland et al., 2020) have been extensively used as vectors for delivery for vaccines, most recently in multiple vaccine candidates for SARS-CoV-2 (Lu et al., 2020; McKay et al., 2020). Bacteria-derived MV can affect diverse biological processes and have emerged as potentially important mediators of pathogen-host interactions (Jan, 2017) and bacteria to bacteria interactions (Caruana and Walper, 2020). It has also been demonstrated that MV represent a strategy for communication between beneficial bacteria and intestinal epithelial cells (Canas et al., 2016). Most of the research has addressed the functions of MV from Gram-negative bacteria and mammalian cells. Gram-positive bacteria, to which most probiotics belong, were initially believed to not produce MV, but during the last decades numerous studies have demonstrated the opposite (Lee et al., 2009; Brown et al., 2015). For instance, it has been described that probiotic bacteria-derived MV could inhibit HIV-1 infection of human tissues (Nahui Palomino et al., 2019), limit the growth of hepatic cancer cells (Behzadi et al., 2017), regulate brain function (Haas-Neill and Forsythe, 2020), and modulate inflammatory responses (Kim et al., 2018; Mata Forsberg et al., 2019; Yang et al., 2019; Choi et al., 2020).

*Limosilactobacillus reuteri* DSM 17938 is one of the most well studied probiotic strains and has been under intense investigation for many years (Walter et al., 2011; Mu et al., 2018). The best-described clinical effect is amelioration of infantile colic, which has been reported in a number of clinical trials (Savino et al., 2010; Szajewska et al., 2012) and confirmed in several meta-analyses (Gutierrez-Castrellon et al., 2017; Sung et al., 2018). The mechanism behind these effects is likely of a complex nature and is, like the etiology of colic, far from being completely understood (Zeevenhooven et al., 2018). However, strain DSM 17938 has in preclinical investigations shown a number of effects that are believed to be important for the relief of colic: several studies have demonstrated its ability to ameliorate inflammation (Hoang et al., 2018; Karimi et al., 2018) and improve the intestinal epithelial barrier function (Karimi et al., 2018); it has also been shown that the strain reduces signaling from the transient receptor potential vanilloid 1 (TRPV1) channel which is a major nociceptive receptor in the intestine (Perez-Burgos et al., 2015); and finally, DSM 17938 has the ability to modulate intestinal motility in an *ex vivo* mouse model (Wu et al., 2013). Interestingly, it has been described that *L. reuteri* DSM 17938 produce MV (Grande et al., 2017) and that those mediate a similar effect on gut motility (West et al., 2020) and possess an immune modulatory activity (Mata Forsberg et al., 2019).

To further increase the knowledge about MV from *L. reuteri*, we have investigated the physicochemical and biological composition of MV derived from two *L. reuteri* strains, and factors affecting MV production. DSM 17938 is a well-described strain with proven probiotic efficacy, and BG-R46 is a related and novel strain of *L. reuteri* with improved *in vitro* properties, making the comparison between the two strains interesting to pursue. We have also investigated the effects of the MV in three types of cell models: intestinal permeability in a Caco-2/HT29-MTX epithelial cell model; immune modulatory effects in a peripheral blood mononuclear cells (PBMC) model; and their ability to dampen activation of TRPV1 in primary rat dorsal root ganglion cells (rDRGs). This study both provides basic knowledge of *L. reuteri* derived MV as well as teaches us about their potential role as effectors involved in probiotic mechanisms, with a focus on infantile colic.

## Materials and methods

### Bacterial strains

Two strains of *L. reuteri* subsp. *kinnaridis* have been used in the study. The first, *L. reuteri* DSM 17938, is a well-studied and widely used strain (Rosander et al., 2008; Li et al., 2021). The second strain *L. reuteri* BG-R46 (also designated DSM 32846) has been obtained after selective breeding of DSM 17938. This strain was cultivated overnight in Man–Rogosa–Sharpe (MRS) broth and thereafter incubated in MRS broth containing 0.5% porcine bile (B8631, Sigma Aldrich) at 37°C for 90 min. The suspension was diluted and plated on MRS agar plates, which were incubated anaerobically at 37°C for 16 h. Among the selected colonies, one isolate was found to have a stable phenotype with significantly smaller colony size and increased secreted 5′ nucleotidase activity compared to DSM 17938. The pure strain was named BG-R46. Both *L. reuteri* DSM 17938 and BG-R46 have been used with permission of BioGaia AB, Stockholm, Sweden.

In addition, *Lacticaseibacillus rhamnosus* strain GG (ATCC 53103) was used as a comparison strain in some of the experiments.

### Cultivation of bacteria, isolation, and basic characterization of extracellular MV

The strains were grown in MRS medium (Oxoid) under different conditions: at 37°C without/with agitation (120 rpm rotation from the beginning of cultivation; the volume size of flask/the volume of culture = 5:1), harvested after 24 or 48 h, separated from the culture broth by centrifugation at 5,000 × *g* for 10 min at 4°C, followed by centrifugation at 10,000 × *g* for 10 min at 4°C, after which any residual cells were removed from the supernatants by filtration using a 0.45 μm pore filter. Supernatants were concentrated using Amicon 100 kDa MWCO (molecular weight cutoff) filter columns. Thereafter the supernatants were centrifuged in a Beckman Coulter Optima L-80XP ultracentrifuge (Beckman Coulter, United States) at 118,000 × *g* at 4°C for 3 h. The supernatants were discarded, the pellets resuspended in PBS buffer and thereafter ultra-centrifuged for a second time (118,000 × *g* at 4°C for 3 h). The pellets were finally suspended in PBS, aliquoted and stored at-70°C. Meanwhile, *L. rhamnosus* GG (LGG) was cultivated in MRS at 37°C without shaking for 24 h. LGG derived MV were used as controls.

The protein and nucleic acid contents of the MV preparations were quantified by using Qubit protein, double stranded DNA and RNA assay kits (Invitrogen) according to the manufacturer’s instructions. All measurements were carried out as three independent experiments.

*L. reuteri* DSM 17938 and BG-R46 bacterial growth was determined by optical density (OD_600_) and colony forming unit (CFU) measurements during cultivation. *L. reuteri* DSM 17938 and BG-R46 were grown in the MRS medium (Oxoid) at 37°C without agitation for 48 h. *L. reuteri* DSM 17938 was also grown in the MRS medium (Oxoid) under the O2 stress condition (120 rpm rotation from the beginning of cultivation; the volume size of the flask/ the volume of the culture=5:1) for 48 h. The optical densities were measured by using a spectrophotometer. CFU were measured after 0, 3, 5, 24 and 48 h cultivation time respectively for each culture. MRS agar (Oxoid) plates incubated anaerobically at 37°C for 48 h were used for CFU determination. The results were expressed as log CFU/ml.

### Morphological characterization of MV by transmission electron microscope (TEM) and scanning electron microscope (SEM)

Ten μl of the MV suspensions were added on a carbon coated grid (2 mm) after dilution with PBS. The grid was maintained at room temperature for 5 min and after excessive liquid was absorbed by filter paper, the MV were negatively stained by 2% Uranyl acetate for 3 min. The MV were rinsed by PBS and then aired before observed and photographed by TEM (H-8100, Hitachi, Tokyo, Japan) at 80–120 kV.

*Limosilactobacillus reuteri* bacterial cells were centrifuged for 20 min at 4000 x *g* at 4°C, washed twice with PBS (pH 7.4), loaded on poly-L-Lysine coated silica wafer substrate, and fixed for 1–2 h at room temperature in the dark with 1% v/v osmium tetroxide in 0.1 M PIPES buffer. After washing three times in PBS buffer, the samples were dehydrated through an ethanol gradient (30, 50, 70, 85, 95, 100%; 15 min each) and treatment with hexamethyldisilazane. Finally, they were sputter coated with gold and photographed by using SEM (Zeiss Gemini 450 II, Zeiss, Oberkochen, Germany).

### Proteomics

Biological triplicates of MV from DSM 17938 and BG-R46 were prepared, pooled and split into three technical replicates. Each MV fraction was isolated from 200 ml cultivations and the final volume were 200 μl per MV fraction. The surface proteome (surfaceome) of the MV fractions were analyzed by the lipid-based protein immobilization (LPI) methodology (Karlsson et al., 2012). Fifty μl was used to fill one LPI channel, and three different channels were used per MV strain. Channel 1–3 consisted of MV from BG-R46 and channels 4–6 from DSM 17938. The samples were immobilized for 45 min, and excess sample fluid was removed from the wells. The channels were then washed with 100 μl PBS, using a manual pipette. Surface shaving (limited proteolysis) of the MV fractions was performed using a trypsin for digestion of the exposed surface proteins. One hundred μl of trypsin solution (20 μg/ml in PBS) was injected into each channel and excess fluid was removed from the wells. Samples were digested for 15 min at RT and the peptides were subsequently collected by eluting 200 μl from each LPI channel. Samples were acidified with 40 μl of 10% formic acid and stored at −20°C.

### Proteomic analysis

Samples were desalted (Pierce peptide desalting spin columns, Thermo Fisher Scientific) according to the manufacturer’s instructions prior to analysis on a QExactive HF mass spectrometer interfaced with Easy-nLC1200 liquid chromatography system (Thermo Fisher Scientific). Peptides were trapped on an Acclaim Pepmap 100 C18 trap column (100 μm × 2 cm, particle size 5 μm, Thermo Fisher Scientific) and separated on an in-house packed analytical column (75 μm × 30 cm, particle size 3 μm, Reprosil-Pur C18, Dr. Maisch) using a gradient from 5% to 80% acetonitrile in 0.2% formic acid over 90 min at a flow of 300 nl/min. The instrument operated in data-dependent mode where the precursor ion mass spectra were acquired at a resolution of 60,000, m/z range 400–1,600. The 10 most intense ions with charge states 2 to 4 were selected for fragmentation using HCD at collision energy settings of 28. The isolation window was set to 1.2 Da and dynamic exclusion to 20 s and 10 ppm. MS2 spectra were recorded at a resolution of 30,000 with maximum injection time set to 110 ms. The data files were searched for identification using Proteome Discoverer version 2.4 (Thermo Fisher Scientific). Since the genome of DSM 17938 is not publicly available, the genome of the parental strain *L. reuteri* ATCC 55730 (Rosander et al., 2008) was used (GenBank BioProject PRJNA30643) to identify and name the proteins. The data was matched against the ATCC 55730 genome using Mascot version 2.5.1 (Matrix Science) as a search engine. The precursor mass tolerance was set to 5 ppm and fragment mass tolerance to 50 mmu. Tryptic peptides were accepted with one missed cleavage and methionine oxidation was set as variable modification. FixedValue was used for PSM validation. The cellular localization of the detected proteins was predicted by using the information generated by Bath et al. (2005). The annotation of the proteins and identification of domains were done by using information from UniProt^1^, MoonProt^2^, and GenBank.^3^

### Nanoparticle tracking analysis

The physicochemical characterization of MV was done by using the Nanoparticle tracking analysis (NTA). MV were diluted with PBS and directly tracked using the NanoSight NS300 system (NanoSight ™ technology, Malvern, United Kingdom). A 488 nm laser beam was used, and three videos of 90 s were recorded of each sample and triplicate histogram were averaged for each sample. Data analysis was performed using the NTA software (version 3.2).

### 5′-nucleotidase (5′NT) activity of MV

5′NT activity from MV was detected by using a 5′-nucleotidase assay kit (Crystal Chem High Performance Assays. USA) according to the manufacturer’s instructions. The level of 5′NT activity from MV which were produced under different conditions and from different strains was quantified. All measurements were carried out as three independent experiments with biological replicates.

### Detection of lipoteichoic acid (LTA) on MV

In the dot-blot assay, MV samples were loaded onto an activated and semi-dry PVDF: polyvinylidene fluoride membrane. The membrane was blocked with TBS-T:Tris-buffered saline with 0.05% Tween 20 (10 mM Tris–HCl, 150 mM NaCl pH 7.5, 0.05% Tween 20), for 1 h at room temperature after which it was incubated for 18 h at 4°C with the primary lipoteichoic acid monoclonal antibody (Thermo Fisher Scientific) at a 1:50 dilution in TBS-T with 1% BSA. The membrane was then washed with TBS-T and incubated with an HRP-conjugated secondary antibody at a dilution of 1:2,000 in TBS-T with 1% BSA, for 1 h at room temperature. After several TBS-T washes, the membrane was developed using an ECL kit (Bio-Rad Laboratories, Inc.). PBS and bacterial cells from *E. coli* were used as negative controls and LTA standards as positive control.

Immunodetection of LTA on MV by confocal microscope Zeiss LSM 780 was performed by staining MV from *L. reuteri* with PKH26 using PKH26 Red Fluorescent Cell Linker Kits for General Cell Membrane Labeling (Sigma-Aldrich) according to the protocol. MV were loaded onto poly-L-lysine coated N.1.5 coverslips and air-dry, followed by fixation with 4% paraformaldehyde in PBS for 10 min at room temperature. Following incubation for 30 min in blocking solution (5% normal goat serum with 0.3% BSA in PBS), the samples were incubated overnight at 4°C with primary LTA monoclonal antibody (Thermo Fisher Scientific) at a 1:50 dilution in blocking solution. The coverslips were washed with PBS and incubated for 1 h at room temperature with a secondary antibody (Abberior STAR 635) at a dilution of 1:100 in blocking solution. After several PBS washes, the coverslips were mounted onto glass slides using Mowiol 4–88 and photographed by confocal microscope Zeiss LSM 780.

### *In vitro* epithelial permeability

The human colon carcinoma cell lines (Caco-2 ATCC HTB-37) and the goblet human colorectal carcinoma cells (HT29-MTX from ECACC) were separately grown in tissue culture flasks in Dulbecco’s Modified Eagle’s Medium (DMEM) supplemented with 10% fetal bovine serum, 1% non-essential amino acids, and 1% penicillin and streptomycin, at 37°C under an atmosphere of 5% CO_2_ with 90% relative humidity. Caco-2 and HT29-MTX cells were grown in 25 cm^2^ tissue culture flasks and split at 80%–90% confluence using 0.25% trypsin and 0.02% ethylenediaminetetraacetic acid (EDTA) solution. The cells were seeded at a density of 6 × 10^4^ cells per 25 cm^2^ flask.

Caco-2 and HT29-MTX cells were seeded on the apical chamber of transwell inserts (Transwell-COL; collagen-coated membrane filters) with 9:1 proportion and grown in 12-well transwell plates (Corning Costar) with a final density of 1 × 10^5^ cells/cm^2^ in each insert. Cells were maintained under the same conditions and allowed to grow for 21 days with medium (0.5 ml on the apical side and 1.5 ml on the basolateral side) that was refreshed every other day to allow the cells to become differentiated. The integrity of the cell layer was determined using two methods: transepithelial electrical resistance (TEER) and determination of fluorescein isothiocyanate-dextran (FITC-dextran) permeability. TEER was measured using the Millicell electrical resistance system (Millipore, Darmstadt, Germany). Each TEER value is the average of 6–9 independent measurements. Wells with TEER values above 250 Ω cm^2^ were used for the permeability studies. Seeded Caco-2/HT29-MTX cells were pre-treated with live *L. reuteri* DSM 17938 cells (cultivated for 24 h) at 100 multiplicity of bacteria (MOB) or MV from *L. reuteri* at 10–200 multiplicity of MV (MOM) for 6 h before challenge with ETEC (enterotoxigenic *E. coli* strain 853/67, known for having a disruptive effect on epithelial integrity; Holmgren, 1973) at 100 multiplicity of infection (MOI) for an additional 6 h. TEER was measured before pre-treatment and challenge with ETEC, followed by measurement every second hour during the entire challenge. To quantify the paracellular permeability of monolayers, 1 mg/ml of 4 kDa FITC-dextran (Sigma) was added to the apical side of the inserts at the start of the challenge with ETEC. Samples from the basolateral compartment were taken after 6 h of incubation. The diffused fluorescent tracer was then analyzed by fluorometry (excitation, 485 nm; emission, 520 nm) using a FLUOstar Omega Microplate Reader (BMG Labtech, Ortenberg, Germany).

### Staining of MV and localization of MV in Caco-2/HT29-MTX cell co-cultures

The MV and control were stained with PKH26 Red Fluorescent Cell Linker Kits for General Cell Membrane Labeling (Sigma-Aldrich). For the control sample, particle-free PBS was used as the input instead of the MV standard. MV and control samples were pelleted by ultracentrifugation (Optima X Series, Beckman coulter, IN, United States) at 190,000 × *g* for 2 h at 4°C. The pellet was gently resuspended in 100 μl PBS and MV were diluted to 6 ml with PBS and placed onto 1.5 ml 0.971 M sucrose cushion. The MV were pelleted by ultracentrifugation at 190,000 × *g* for 2 h at 4°C. The pellets were gently washed with PBS and resuspended in PBS, followed by transfer to an Amicon 10 kDa MWCO filter column that was repeatedly centrifuged at 3,000 x *g* for 40 min at 4°C to reduce the volume to 0.5–1 ml. Caco-2/HT29-MTX co-cultured cells were grown on transwell filters in 12-well tissue culture plates as described above. On day 21, upon which the monolayer reached polarization, cells were either apically treated with stained MV for 6 h or left untreated. Filters were fixed in 4% paraformaldehyde for 15 min at 4°C and permeabilized with 0.2% Triton-X-100 for 15 min at RT. Membranes were rinsed with PBS. The membrane with cells was then incubated in a 3% BSA solution (Sigma Aldrich) for 1 h at RT. Mouse monoclonal anti-ZO-1 (diluted at 1:100; N-term; Invitrogen, Carlsbad, CA) was applied as primary antibody and Alexa Fluor 488 goat anti-mouse (green) as the secondary antibody (diluted at 1:200; Invitrogen). The nuclei were stained with DAPI (Invitrogen). Images were acquired using laser scanning confocal microscopy (Zeiss LSM 780 with a 63× objective; Zeiss ZEN software; Zeiss, Oberkochen, Germany).

### PBMC stimulations

Healthy, anonymous, adult volunteers were included in this study, which was approved by the Regional Ethics Committee at the Karolinska Institute, Stockholm, Sweden [Dnr 2014/2052–32]. All methods were carried out in accordance with approved guidelines and all study subjects gave their informed written consent. Venous blood was collected and diluted 1:1 with RPMI-1640 cell culture medium supplemented with 20 mM HEPES (HyClone Laboratories, Inc.). Peripheral blood mononuclear cells (PBMC) were isolated by Ficoll–Hypaque (GE Healthcare Bio-Sciences AB) gradient separation. The isolated PBMC were washed and resuspended in freezing medium containing 40% RPMI-1640, 50% fetal bovine serum (FBS; Sigma Aldrich) and 10% DMSO (Sigma-Aldrich), frozen gradually at-80°C in freezing containers (Mr. Frosty, Nalgene Cryo 1°C; Nalge CO.) and finally stored in liquid nitrogen until used in assays.

PBMC were thawed, washed, and stained with Trypan blue followed by live cell counting using a 40x light microscope. Cells were resuspended in cell culture medium containing RPMI-1640 supplemented with HEPES (20 mM), penicillin (100 U/ml), streptomycin (100 μg/ml), L-glutamine (2 mM; all from HyClone Laboratories, Inc.) and FBS 10% (Sigma Aldrich) at a final concentration of 1×10^6^ cells/ml. Cells were seeded in flat bottomed 96-well cell culture plates at 2.5×10^5^ cells/well and incubated for 48 h at 37°C with 5% CO_2_ atmosphere. *Staphylococcus aureus* (*S. aureus*) 161:2 (Haileselassie et al., 2013) -cell free supernatant (CFS) was used as a stimulus at 2.5% (v/v) and lactobacilli-MV were used at a MV-to-cell ratio of 500:1. Finally, the cell culture supernatants were collected by centrifugation and stored at-20°C.

### Enzyme-linked immunosorbent assay

Secreted levels of the cytokines IL-1β, IL-6, IFN-γ and TNF-α in cell culture supernatants were determined using sandwich ELISA kits (MabTech AB) according to the manufacturer’s instructions. Absorbance was measured at a wavelength of 405 nm using a microplate reader (Molecular Devices Corp.) and results analyzed using SoftMax Pro 5.2 rev C (Molecular Devices Corp.).

### TRPV1 antagonistic potential in primary rDRGs

The experimental procedures were conducted under ethical permit no. 76-2013 and in accordance with European and Swedish animal welfare regulations. Cultures of primary rat dorsal root ganglia cells derived from 6-week-old male Sprague–Dawley rats were obtained by microsurgical dissection, performed at the University of Gothenburg, after which the rDRGs were grown in 384-well plates. The cells were stained with the Ca^2+^ indicator Ca5 (FLIPR Calcium 5 Assay Kit, Molecular devices, CA, USA) on the experiment day to measure intracellular calcium ion flux. MV suspended in culture medium containing Neurobasal A (Gibco, Camarillo, CA, USA) supplemented with supplement B27 (Invitrogen, Grand Island, NE, USA) and Glutamax (Thermo Fisher Scientific, Agawam, MA, USA) were added in 6 concentrations (diluted in steps 1:3) 1 h before adding the agonist capsaicin. The highest concentration of vesicles was 10% of the stock which corresponded to approximately 10^9^ particles/ml. Measurements were taken in the Cellaxess Elektra discovery platform where the calcium probe intensity was measured continuously. Experiments were conducted in three replicates at two or three separate time points, *n* = 9 for DSM 17938 24 h, DSM 17938 48 h, BG-R46 48 h, LGG 24 h, and *n* = 6 for BG-R46 24 h. AMG517, a known TRPV1 antagonist, were used as control substance to verify the antagonistic effect.

### Statistical analysis

Data are generally expressed as means and standard deviations, except for the immunological data which is displayed as median with interquartile range. The difference among groups was analyzed by one-way analysis of variance (ANOVA) unless stated otherwise. The significant difference was set at *p* < 0.05. In the epithelial integrity experiment comparing bacterial cells and MV, Welch’s ANOVA was used. A two-way ANOVA was used for the FITC-measurements in the epithelial integrity experiment comparing different concentrations of MV. For naïve PBMC cytokine secretion, Mann–Whitney statistical test was used. Wilcoxon matched pairs signed rank test was performed for the relative concentrations of TNF-α and IFN-γ. A mixed effects model was used in the TRPV1-model.

## Results

### Production parameters and strain features affect MV characteristics

**Figure 1:**
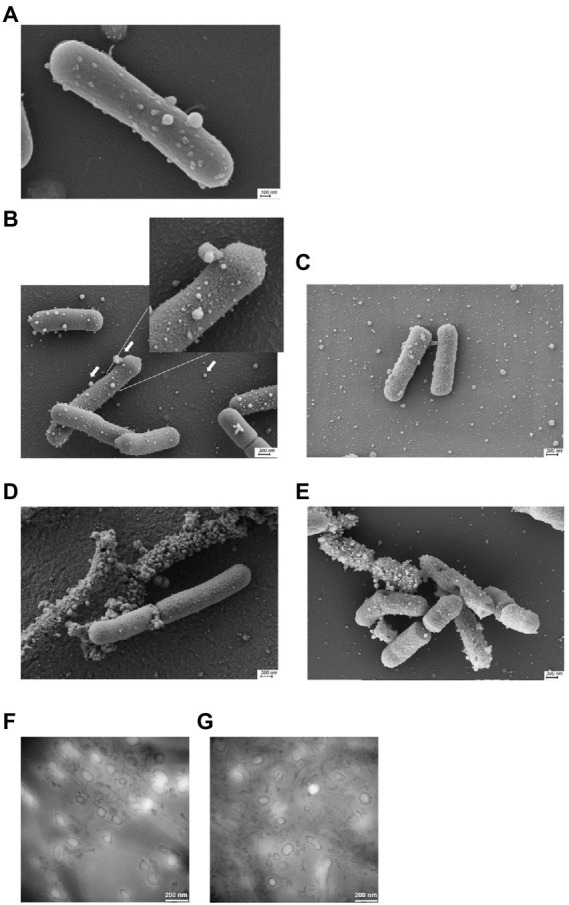
Scanning electron microscopy images of *L. reuteri* cultivated under different conditions. *L. reuteri* DSM 17938 cultivated in MRS for 24 h with extracellular membrane vesicles of varying sizes budding of the bacterial surface **(A)**. *L. reuteri* DSM 17938 after 24 h with **(D)**/without O_2_ stress condition **(B)** and 48 h **(E)** of cultivation; BG-R46 after 24 h cultivation **(C)**. A representative example of three independent experiments is shown. Magnification 40k. Arrows indicate MV budding from the cell surface and released MV. Transmission electron microscopy negative staining analysis of *L. reuteri* DSM 17938 extracellular membrane vesicles isolated after 24 h **(F)** and 48 h **(G)** of cultivation.

**Figure 2:**
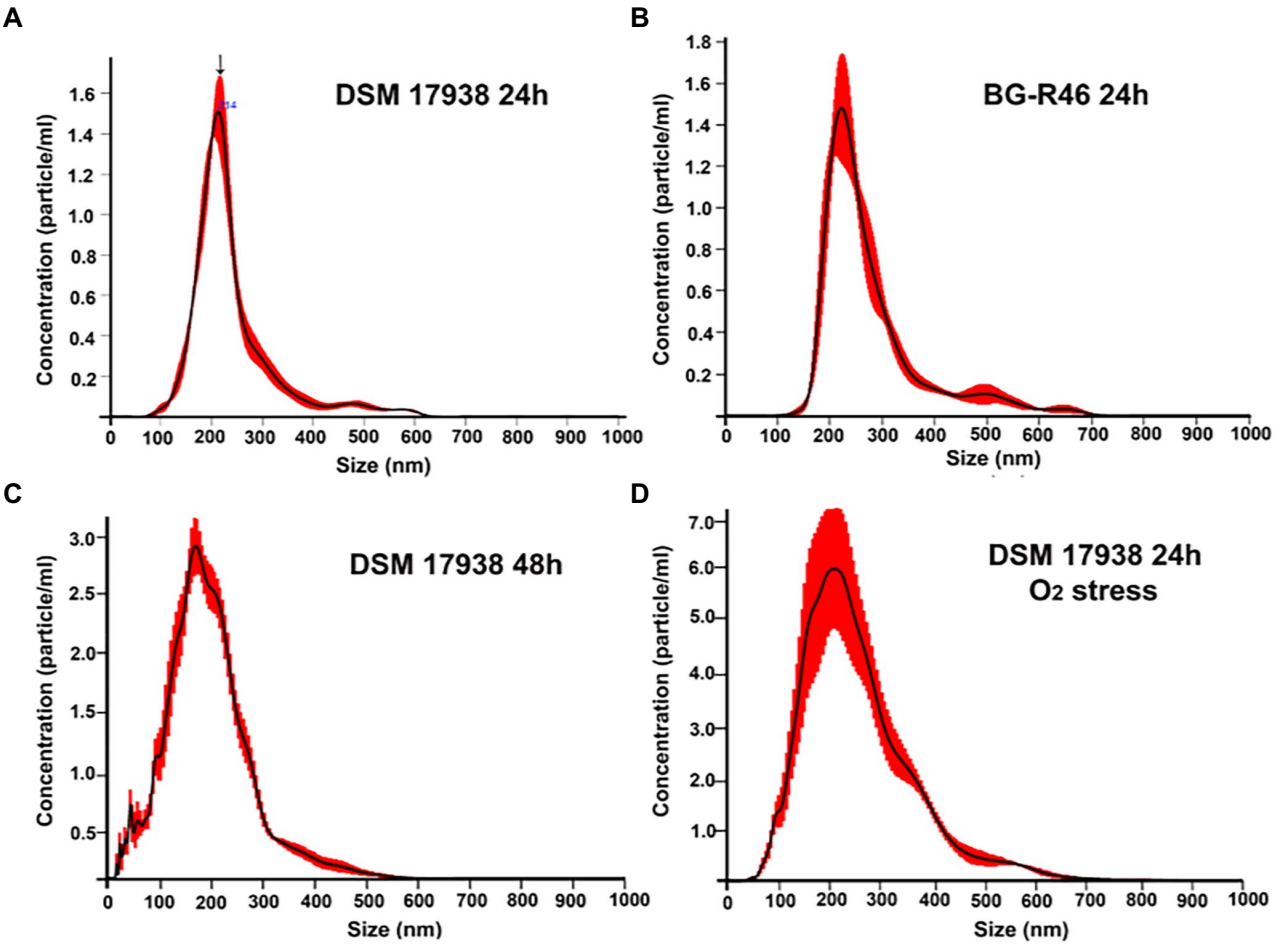
Physicochemical characterization of *L. reuteri* derived MV through NanoSight tracking analysis (NTA). The nanoparticle tracking distribution of *L. reuteri* DSM 17938 derived MV after 24 h, 48 h, under O_2_ stress of cultivation and isolated from BG-R46 were shown in **(A,C,D,B)** individually. Figures are representative of three independent triplicates. The size distribution represents the wide distribution of MV for each MV prep. Arrow shows the mean peaks of particles at 214 nm **(A)**. Figure 2A was originally published in Scientific Reports (Mata Forsberg et al., 2019).

To increase the general knowledge of MV from *L. reuteri* DSM 17938, we initially cultivated DSM 17938 under standard conditions for 24 h. Cultivation time affected the survival and appearance of the bacterial cells, and after 24 h of cultivation most bacteria were alive, but the viability had dropped approximately 30 times after 48 h (Supplementary Figure S1). The bacterial cells were imaged by SEM showing a large number of budding vesicles on the cell surface (Figure 1A). As the next step we studied the production of MV during different culture conditions and performed a physiochemical characterization of the MV. The production was affected by the culture conditions and both prolonged cultivation time (48 h) and oxygen stress resulted in a 16-fold increase of MV (Table 1). We also investigated the strain *L. reuteri* BG-R46, which was found to produce approximately the same amount of MV as DSM 17938. We discovered that the MV contained protein, RNA and DNA. The protein concentrations of *L. reuteri* DSM 17938 derived MV from 48 h-cultures and oxygen stressed bacteria were 2-fold higher than those from the 24 h-cultures (*p* < 0.05; Table 1). SEM analyses revealed that the bacterial cells from both strains were intact after 24 h and multiple vesicles per cell were observed (Figures 1B,C). However, both the extended cultivation time (48 h) and the oxygen stress led to disintegration of bacterial cells (Figures 2D,E). MV appeared on the bacterial surface and were also released from the bacterial cells (Figures 1A–E). Analysis of the MV by TEM showed that they had a broad particle distribution, polymorphic structure, and spherical shape (Figures 1F,G). The size-heterogeneity of the vesicles was confirmed with nanoparticle tracking analysis, which revealed a wide size distribution (MV’s diameter from 20 nm up to 500 nm) of the isolated MV (Figure 2). The size profiles of MV from the two strains were quite similar after 24 h, but there were broader size distributions of MV from 48 h and after O_2_ stress. The Nanoparticle Tracking Analysis (NTA) analysis detected the presence of larger particles which may be aggregates of MV (Figure 2).

**Table 1:** Quantification of *L. reuteri* derived extracellular membrane vesicles (MV) and their content of DNA, RNA, and protein.

MV content	DSM 17938 24 h	BG-R46 24 h	DSM 17938 O_2_ stress 24 h	DSM 17938 48 h
MV conc. (particles/ml)	3.0 ± 1.9 × 10^9a^	3.0 ± 2.7 × 10^9a^	5.3 ± 1.1 × 10^10b^	4.9 ± 2.6 × 10^10b^
dsDNA (μg/ml)	0.51 ± 0.3^a^	0.54 ± 0.1^a^	1.26 ± 0.4^a^	0.68 ± 0.01^a^
RNA (μg/ml)	2.27 ± 0.8^a^	2.65 ± 0.25^a^	3.46 ± 0.2^a^	3.28 ± 0.4^a^
Protein (μg/ml)	183.5 ± 30^a^	216.25 ± 28^a^	506 ± 20^b^	462.5 ± 50^b^

Value with the same letter is not significantly different from one another determined by one-way analysis of variance (ANOVA), *p* < 0.05. All values are mean (*n* = 3), error bars are SD (*n* = 3). ^a,b^indicate statistical differences between samples.

### Proteome analysis and surface characteristics of MV

**Figure 3:**
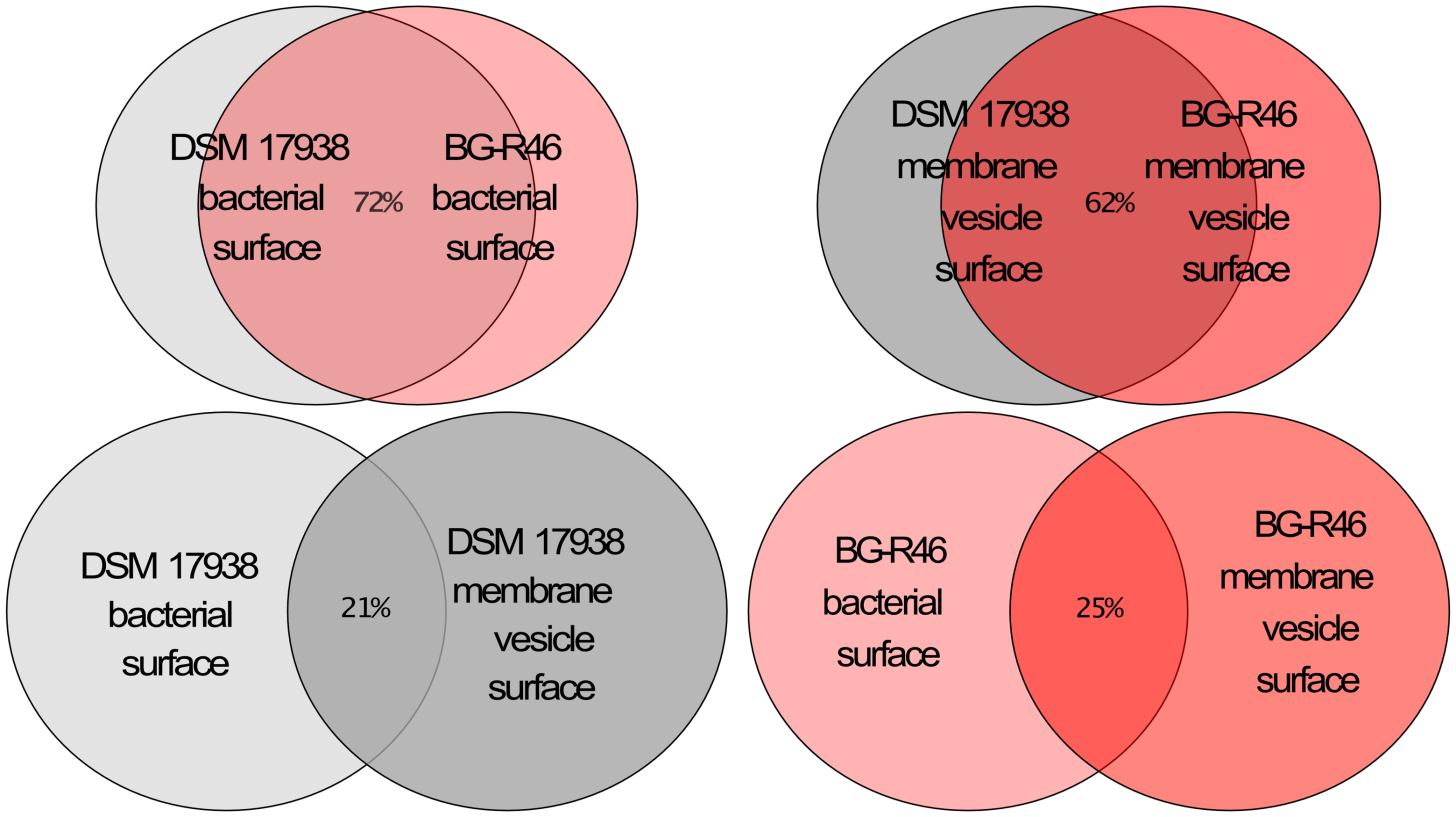
Venn diagram displaying a comparison of the 100 most abundant proteins on the bacterial surfaces and the membrane vesicle surfaces of DSM 17938 and BG-R46. The proteomic analysis was performed by using a trypsin mediated surface shaving, Lipid-based Protein Immobilization method. The color key is gray = DSM 17938 and red = BG-R46, light color indicate bacterial surface, dark color indicate membrane vesicle surface.

To further investigate the protein exposed on the MV, we analyzed MV isolated from both DSM 17938 and BG-R46 using liquid chromatography tandem mass spectrometry (LC–MS/MS). More than 800 proteins were identified (data not shown), and the vast majority of the proteins with highest #PSM and #peptides scores were predicted to be secreted (Supplementary Table S1). It means that they normally would be predicted to be localized on the bacterial cell surface or being released from the bacteria, and many of those proteins are tentatively involved in host-bacterial interactions (Supplementary Table S1). Several of the proteins are predicted to be involved in adhesion to cells and mucus; a LPXTG domain protein with a Rib/alpha-like repeat (HMPREF0538_20063), a MucBP protein (HMPREF0538_20356), an YSIRK signal peptide protein with 9 Rib/alpha-like repeats (HMPREF0538_20775–20,774), and the earlier described collagen/mucus binding protein CnBP (HMPREF0538_21501). In addition, several moonlighting proteins were detected, most of which were predicted to be involved in adhesion. The MV preparations also contained proteins predicted to be involved in production of extracellular polysaccharides (EPS). One of the most abundant proteins was a dextran sucrase (HMPREF0538_20764) and several proteins expressed from a big EPS operon were detected (HMPREF0538_20363, HMPREF0538_20382, HMPREF0538_20383). Another abundant protein detected in the MV preparations was a LPxTG anchored 5′-nucleotidase (5′NT; HMPREF0538_20056). This enzyme converts adenosine monophosphate (AMP) to adenosine and this activity was confirmed with an enzymatic assay (Table 2). The 5′NT activities of MV from 48 h (DSM 17938) and oxygen stressed condition were more than 15-fold higher compared to the activity at 24 h (DSM 17938) which is in line with the MV concentration ratios described above. Interestingly, BG-R46 derived MV had more than 7-fold higher 5′NT activity compared to the corresponding DSM 17938 preparation, although they had the same concentration of MV. 5′NT activity from *L. rhamnosus* GG (LGG) derived MV was not detectable. Proteins involved in cell wall modulation was another abundant group. Both proteins involved in synthesis of peptidoglycan (penicillin-binding proteins; Pbp2b and Pbp1a) and degrading peptidoglycan (HMPREF0538_20363, HMPREF0538_21064) were detected. Also, a protein involved in LTA biosynthesis (HMPREF0538_21428) was found. Even though the proteomic approach used here was for general protein identification, i.e., not quantitative proteomics, some proteins seem to be present to a greater extent (#peptides and #PSMs) in DSM 17938 vesicles than in BG-R46 vesicles. Interestingly, one of those proteins were the above-mentioned 5′NT. The top 100 most abundant proteins sorted by #PSM showed a 72% overlap between DSM 17938 and BG-R46 bacterial surfaces. Similarly, the overlap between the two strains MV were 62%. Meanwhile, among the top 100 proteins sorted by #PSM, there was only 21% identity between DSM 17938 bacterial surface and DSM 17938 membrane vesicle surface and 25% between BG-R46 bacterial surface and BG-R46 membrane vesicle surface (Figure 3). This indicates that there is a large difference between what is present on the bacterial and MV surfaces.

**Table 2:** 5′-nucleotidase activity of MV which was isolated from *L. reuteri* under different cultivation conditions.

MV	24 h DSM 17938	24 h BG-R46	48 h DSM 17938	24 h DSM 17938 O_2_ stress	24 h LGG
5′-nucleotidase activity (U/1 × 10^12^ MV)	3.8 ± 0.6^a^	27.3 ± 4.1^b^	3.2 ± 0.6^a^	3.5 ± 0.4^a^	Not detectable
5′-nucleotidase activity (U/L)	11.4 ± 1.2	82 ± 11	169.9 ± 6.8	170.3 ± 9.6	Not detectable

24 h DSM 17938, 48 h DSM 17938 denote MV from *L. reuteri* DSM 17938 harvested at 24 and 48 h; 24 h DSM 17938 O_2_ stress denotes MV from *L. reuteri* DSM 17938 cultivated under O_2_ stress and harvested at 24 h; 24 h BG-R46 denotes MV from *L. reuteri* BG-R46 and harvested at 24 h. Values with different letters are significantly different determined by one-way analysis of variance (ANOVA), *p* < 0.05. Data are given as means three independent measurements. Error bars are SD (*n* = 3).

**Figure 4:**
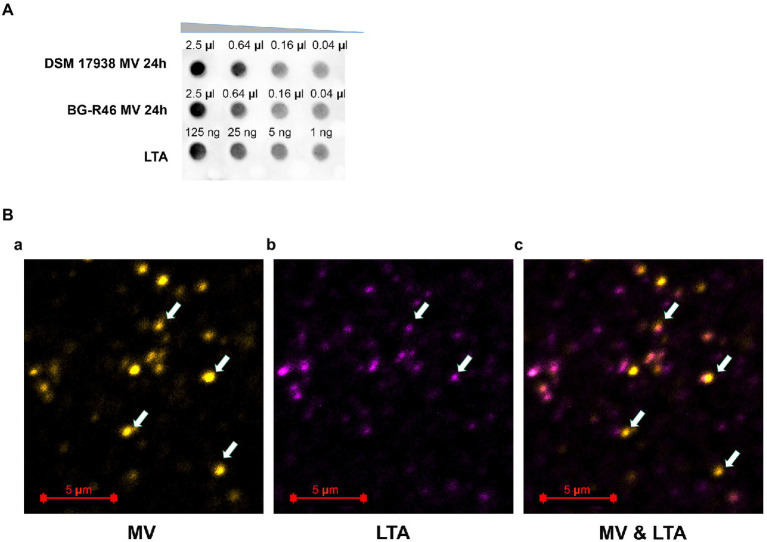
Lipoteichoic acid (LTA) presents on *L. reuteri* DSM 17938 and BG-R46 derived MV. **(A)** Dot-blot assay using the primary antibody against lipoteichoic acid (Thermo Fisher Scientific). The samples were loaded onto an activated and semi-dry PVDF membrane with different amount. The concentrations for MV from *L. reuteri* DSM 17938 and BG-R46 were 4.4×10^9^ particles/ml. **(B)** Confocal immunofluorescence microscopy images of MV from *L. reuteri* DSM 17938. MV were stained by PKH26 (yellow color) **(a)** and LTA were immunolabeled by a primary antibody against lipoteichoic acid (Thermofisher) and a secondary antibody (Abberior STAR 635) which is seen as violet color **(b)**; **(c)** is the superimposing of these two labels. The arrow indicates the MV in the images. MV aggregates were also observed in these images (big dots).

We also investigated if LTA was present on MV. The dot blot assay showed that MV derived from both *L. reuteri* DSM 17938 and BG-R46 carried LTA (Figure 4A). Also, according to confocal immunofluorescence microscope images, LTA was detected on MV (Figure 4B). All the evidence indicated that *L. reuteri* DSM 17938 and BG-R46 derived MV have LTA exposed on the surface. LTA was used as positive control (Figure 4A) and *E. coli* cells as a negative control (data not shown). A schematic image of MV with its tentatively bioactive molecules is presented in Supplementary Figure S2.

### *Limosilactobacillus reuteri* and its derived MV protect epithelial barrier integrity from the detrimental effect of enterotoxigenic *Escherichia coli* (ETEC)

**Figure 5:**
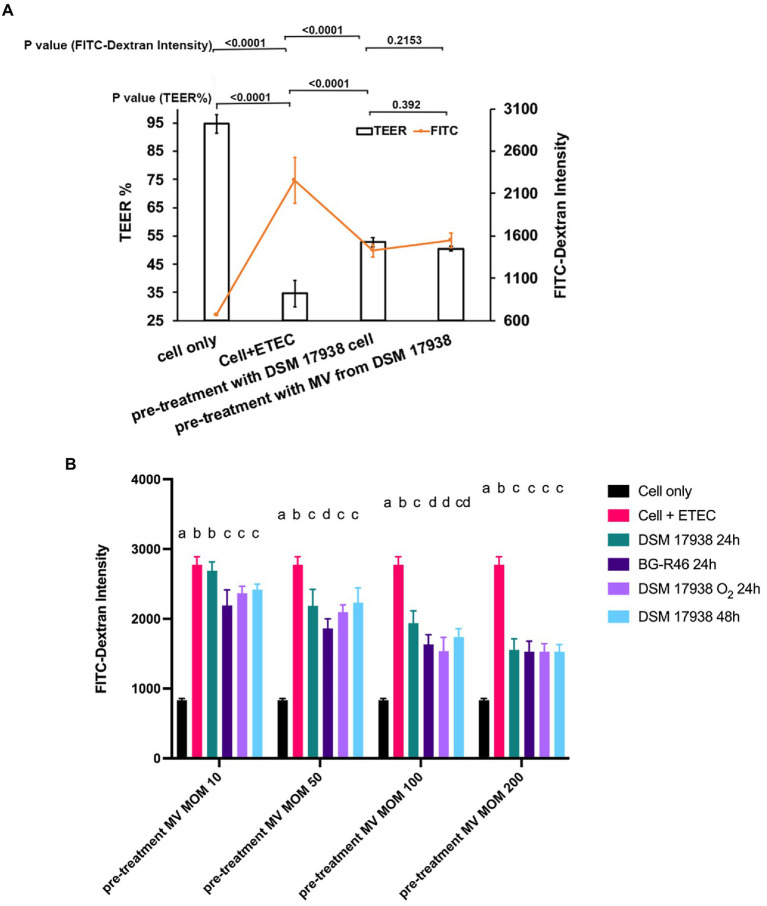
Effect of *L. reuteri* derived MV on the barrier integrity of monolayers treated with ETEC. The Caco-2/HT29-MTX co-cultures were grown on transwell filters for 21 days. **(A)** Polarized monolayers with/without pre-treatment of *L. reuteri* DSM 17938 bacteria cells and derived MV at multiplicity of bacteria/MV of 100:1 and 200:1 for 6 h. After 6 h pre-treatment, the monolayer was infected with ETEC at multiplicity of infection (MOI) 100 for 6 h. TEER was measured in ohms and corrected for the resistance of the blank filters and for the membrane area and expressed as percentage of the starting value (before pre-treatment with MV). The fluorescence intensity is from the fluorescein isothiocyanate (FITC)-dextran which was detected from the basolateral pole. Data are given as means of six independent seedings. Statistical analysis by one-way analysis of variance (ANOVA), *p* < 0.05. **(B)** Polarized monolayers with/without pre-treatment of MV which were isolated from *L. reuteri* under different cultivation conditions. The fluorescence intensity is from the FITC-dextran which was detected from basolateral pole of each sample. Columns with different letters are significantly different (*p*<0.05).. Statistical analysis by two-way ANOVA. Error bars are SD (*n* = 9).

Next, we wanted to investigate whether *L. reuteri* derived MV could protect epithelial barrier integrity of monolayers of cultivated epithelial cells. We used a model with a mix of Caco-2 and HT29-MTX cells. The cells were exposed to enterotoxigenic *E. coli* (ETEC) which induced a strong reduction in transepithelial electrical resistance (TEER), but pre-treatment with either *L. reuteri* bacterial cells or MV from *L. reuteri* provided a protective effect against this reduction (Figure 5A). Furthermore, a FITC-dextran flux experiment demonstrated that both MV and bacterial cells decreased the ETEC induced leakage of the macromolecule. When we pre-treated the Caco-2/HT29-MTX co-cultures with MV (multiplicity of MV per epithelial cell of 200:1), the leakage caused by ETEC decreased approximately by 65% (Figure 5B).

We also compared the effects of the different MV preparations, and already at MOM 10 all variants except DSM 17938 24 h gave a protective effect (Figure 5B). At MOM 50–200 all variants protected. MV from *L. reuteri* BG-R46 gave a significantly better protection than DSM 17938 24 h at all doses except MOM 200 (Figure 5B). However, all MV gave the same protection level at MOM 200. The MV preparations protected the epithelial cells against ETEC challenge in a dose-dependent manner.

**Figure 6:**
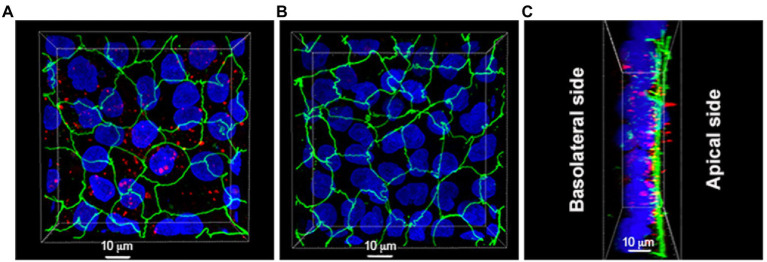
Localization of MV derived from *L. reuteri* DSM 17938 in Caco-2/HT29-MTX cells. **(A)** Caco-2/HT29-MTX cells were pretreated with MV for 6 h. **(B)** Caco-2/HT29-MTX cells were pretreated with PBS buffer which was used for resuspension of MV for 6 h. **(C)** Most of MV were taken up by Caco-2/HT29-MTX cells. Unbound vesicles were washed away prior to the experiment. Images are representatives of four separate experiments, color key: blue = nuclei, green = ZO-1, red = MV.

We further investigated the interaction between MV and Caco-2/HT29-MTX cells by using confocal microscopy. Interestingly, MV were taken up and could be detected inside the epithelial cells or potentially having a paracellular location (Figure 6; Supplementary Video S1).

### MV from *Limosilactobacillus reuteri* strains modulate cytokine production from human PBMC cultures

**Figure 7:**
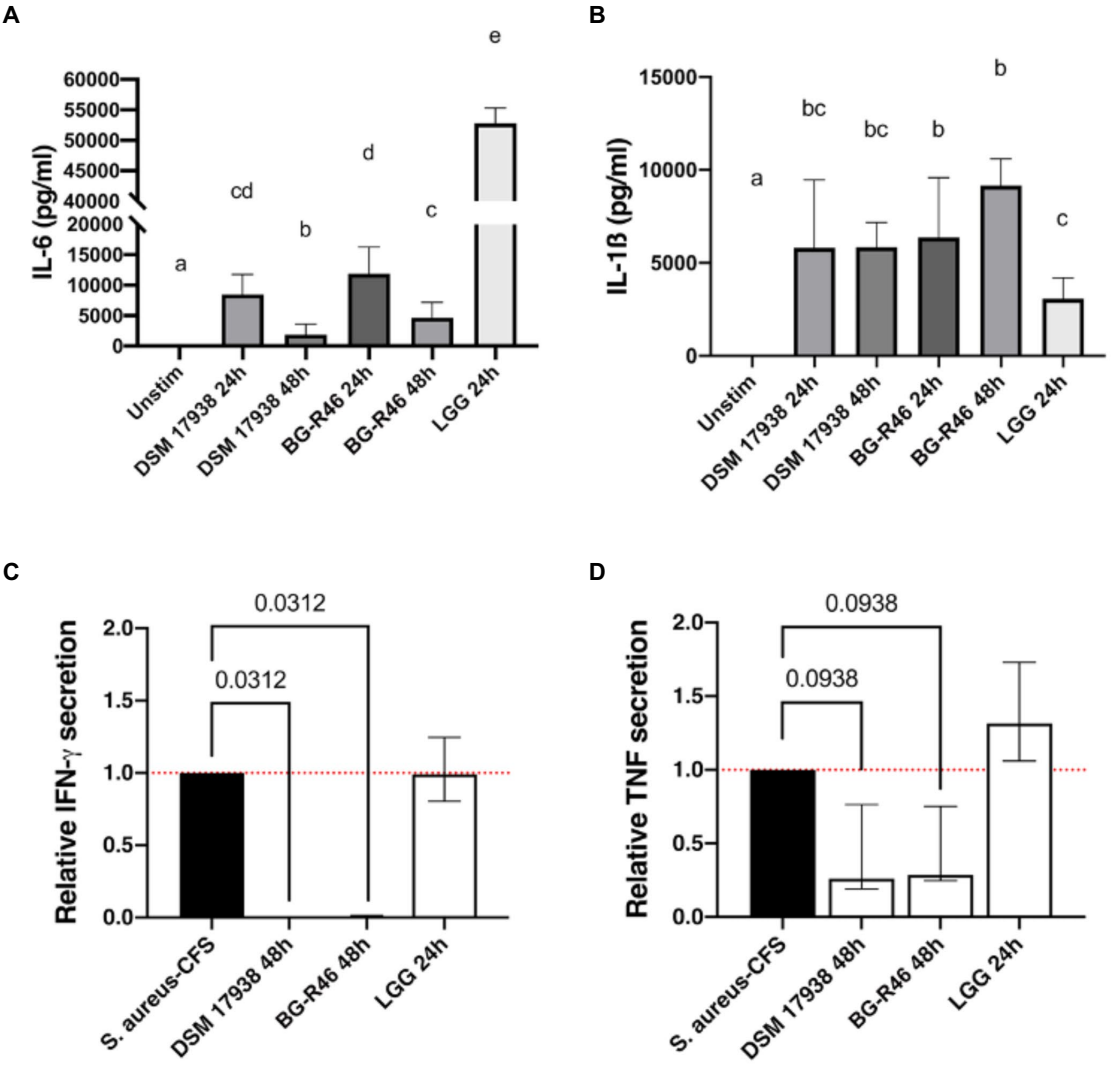
Immunomodulatory effects of *L. reuteri* DSM 17938, BG-R46 and LGG derived MV in PBMC. Secretion of cytokines IL-6 **(A)** and IL-1β **(B)** in naïve PBMC after incubation with MV derived from DSM 17938, BG-R46 and LGG. PBMC were cultured with MV for 48 h before quantification. Bar plots show median with interquartile range. Bars with different letters are significantly different (*p*<0.05). Statistical analysis by Mann–Whitney test, *n* = 6. Relative secretion of IFN-γ **(C)** and TNF-α **(D)** in *S. aureus* stimulated-PBMC in response to incubation with MV derived from *L. reuteri* DSM 17938 and BG-R46 and LGG. PBMC were cultured with MV for 48 h before quantification. Quantification of IFN-γ and TNF-α levels from PBMC stimulated with *S. aureus* were set to 1. Statistical analysis by Wilcoxon matched pairs signed rank test, *n* = 6.

Subsequently, we investigated how the MV preparations affected the stimulatory potential of peripheral blood mononuclear cells (PBMC). The immune cells were cultured for 48 h in the presence of MV isolated from lactobacilli grown for 24 or 48 h. Interestingly, DSM 17938, BG-R46 and LGG derived MV harvested after 24 h induced interleukin (IL)-6 and IL-1β. MV isolated from DSM 17938 and BG-R46 grown for 48 h induced to a lower extent secretion of IL-6, while IL-1β was not affected by cultivation time (Figures 7A,B). We further focused on MV derived after 48 h to investigate the ability of MV to regulate cytokine responses induced by a potential pathogen. MV were added to *S. aureus* cell free supernatant (CFS) challenged PBMC cultures followed by cytokine measurement. Addition of *L. reuteri*-derived MV to *S. aureus*-stimulated PBMC significantly blocked secretion of the proinflammatory cytokine IFN-γ (Figure 7C) and reduced the secretion of TNF-α (Figure 7D), whereas the MV derived from LGG did not decrease the *S. aureus*-induced secretion of IFN-γ and TNF-α.

### MV from *Limosilactobacillus reuteri* antagonize the TRPV1 receptor

**Figure 8:**
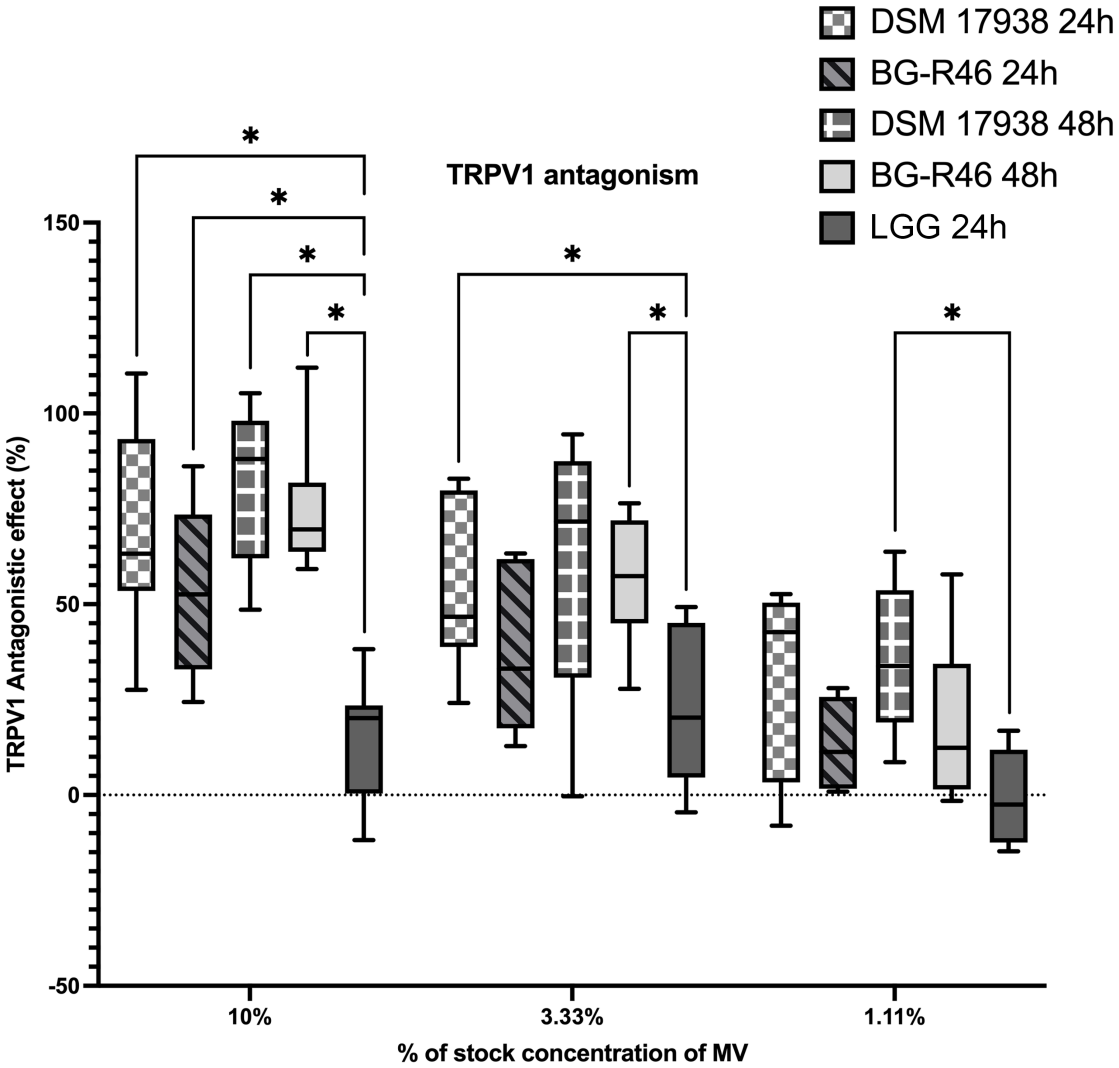
TRPV1 antagonistic effect of extracellular membrane vesicles in primary rat dorsal root ganglion cells (rDRGs). Primary rDRGs were incubated with membrane vesicles in three different concentrations (10%, 3.33%, 1.11%) for 1 h before the experiment. Capsaicin was added and Ca^2+^ was measured and antagonistic response was calculated. Data show that *L. reuteri* DSM 17938 and BG-R46 derived MV antagonize TRPV1 in a dose dependent manner. Box plot with min to max and average value representation. DSM 17938 24 h denote MV from *L. reuteri* DSM 17938 24 h; DSM 17938 48 h denote MV from *L. reuteri* DSM 17938 48 h; BG-R46 24 h denotes MV from *L. reuteri* BG-R46 harvested at 24 h; BG-R46 48 h denotes MV from *L. reuteri* BG-R46 harvested at 48 h. Box plot with min to max and average value representation. *N* = 9 for DSM 17938, DSM 17938, BG-R46 48 h. *N* = 6 for BG-R46 24 h. Asterix * indicates *p* < 0.05.

TRPV1 has been suggested to be involved in pain perception in infantile colic. Therefore, we studied the effect of MV from *L. reuteri* in a rat dorsal root ganglion cells TRPV1 model. The antagonistic effect in % was calculated from the fluorescence intensity of the Ca^2+^ indicator Ca5. A dose dependency in TRPV1 antagonism was observed in response to MV preparations from both DSM 17938 and BG-R46, diluted 10, 30 and 90 times. Control vesicles derived from LGG did not exhibit this antagonistic effect. These results showed that MV from both DSM 17938 and BG-R46 exhibit a significantly stronger antagonistic effect on the TRPV1 receptor compared to the LGG vesicles (Figure 8).

## Discussion

We isolated MV from *L. reuteri* cultivated under different conditions and this resulted in differences in yield and different morphologies (Figure 1). The yield increased with longer cultivation time and in response to oxygen stress and this correlated with fewer live bacteria (Supplementary Figure S1). In concordance with that, more vesicles appeared on the surface of partly degraded cells (Figures 1D,E). Membrane fragments from dead cells can vesicularize and consequently increase the amount of MV, a phenomenon that previously has been described (Toyofuku et al., 2017). However, the SEM analysis (Figure 1) showed vesicles budding on the surface of intact *L. reuteri* cells, indicating that there is a MV biogenesis mechanism that is independent of cell death, a process that also have been reported by others (Brown et al., 2015; Orench-Rivera and Kuehn, 2016). Altogether, this indicates the existence of subpopulations of MV, and future studies could reveal if those have different content and functions.

Although the studies regarding MV production in Gram-positive bacteria has intensified, the mechanisms of vesiculogenesis and transport through the cell wall remains poorly understood. It has been discussed if cell wall modifying enzymes play a role in the MV release through the Gram-positive cell wall (Brown et al., 2015; Toyofuku et al., 2017) and the results from the proteome analysis support this hypothesis. First, MV from *L. reuteri* DSM 17938 carry several peptidoglycan-degrading enzymes (Supplementary Table S1), which potentially could generate channels through the cell wall. Through these channels, cytoplasmic membrane material might be forced by turgor pressure to protrude into the extracellular space and thereafter released as MV. Furthermore, the MV also carry several transpeptidases (Supplementary Table S1) that possibly could be involved in healing of the cell wall after the vesicular release. Thus, the controlled release of MV from *L. reuteri* DSM 17938 could potentially be facilitated by both peptidoglycan degrading and biosynthesis enzymes.

Previous studies have shown that MV can carry a wide range of cargo, including DNA, RNA and proteins (Habier et al., 2018), potentially having the ability to deliver combinatorial information to different types of cells in their microenvironment (Skog et al., 2008). It has been shown that MV from pathogenic *S. aureus* carry RNA and DNA, which are protected from degradation, and that these molecules play an important role in virulence and immune modulation through their transmission of signals to host cells (Lee, 2019; Rodriguez and Kuehn, 2020). MV from *L. reuteri* contain both RNA and DNA and detailed investigations of the nucleic acids content of *L. reuteri* derived MV will be a subject for future studies. As already mentioned, MV preparations from *L. reuteri* also contained proteins (Table 1) and after performing a proteome analysis it was concluded that they represent a wide array of functions (Supplementary Table S1). Interestingly, a majority of the most abundant proteins were predicted to be localized on the cell surface, which differ from other studies of MV from lactobacilli and other potentially probiotic bacteria (Al-Nedawi et al., 2015; De Rezende Rodovalho et al., 2020; Hu et al., 2021). Besides the cell wall biogenesis proteins discussed above, several of the surface proteins are predicted to be involved in host–microbe interactions. A key feature for many bacteria is adhesion to surfaces and this is believed to be true also for a probiotic bacterium (Muscariello et al., 2020). Binding to the mucosal surface will both prolong the persistence in the intestine and enable a closer contact with enterocytes and immune cells with which the bacterium could interact. Some proteins to highlight are (i) the collagen/mucus binding protein CnBP that has been shown to be an important adhesion factor for *L. reuteri* (Roos et al., 1996; Velez et al., 2007); (ii) the large Rib motif containing protein Lr1694, a type of protein that has been described to induce protective immunity and promote binding to human epithelial cells of streptococci (Stalhammar-Carlemalm et al., 1999); and (iii) Lr1612 a large MucBP protein, containing motifs that have shown to be involved in adhesion to mucus and cells (Roos and Jonsson, 2002; Van Tassell and Miller, 2011). It is intriguing that also the MV from *L. reuteri* carry those adhesion proteins, and it both supports the results from the cell models (e.g., Figure 6) and open for further studies of intimate interactions between MV and host cells. Another set of interesting proteins present on the surface of the membrane vesicles are the relatively high number of moonlighting proteins which has been described in other bacteria, i.e., proteins with dual functions intracellularly and extracellularly (Kainulainen and Korhonen, 2014). The vesicles possess a number of these and the fact that they are found with a surface shaving-based proteomics method emphasizes their moonlighting function. Among the moonlighting proteins we found enolase, glyceraldehyde-3-phosphate dehydrogenase, pyruvate kinase, glucose 6-phosphate isomerase, elongation factor G and endopeptidase O. These proteins have been shown to be highly involved in host interactions by facilitating mucus- and epithelial cell- binding functions extracellularly (MoonProt^4^). Similarly, moonlighting proteins have previously been shown to be in high abundance on outer membrane vesicles from Gram-negative bacteria (Dineshkumar et al., 2020).

Already in 1967 it was observed that extracellular products from pathogen bacteria could be engaged in immunomodulatory activities (Chatterjee and Das, 1967). It is also well known that pathogen derived MV can be detrimental and disturb the GI tract homeostasis (Ismail et al., 2003; Bielaszewska et al., 2013). On the contrary, MV from some microbes may help to maintain the homeostasis of the GI tract. MV from both probiotic *E. coli* Nissle 1917 and ECOR63 have been found to upregulate the expression of tight junction proteins and by that strengthen the barrier of intestinal epithelial cells (IECs; Alvarez et al., 2016). It has been reported that *L. reuteri* protect intestinal epithelial monolayers by downregulating expression of IL-6 and TNF-α, induce cytoprotective heat shock proteins (HSP), increase expression of tight junction protein and decrease the adhesion of pathogens (Liu et al., 2015; Karimi et al., 2018). In the current study, it was shown that *L. reuteri* bacterial cells and also their derived MV can protect the epithelial barrier integrity of Caco-2/HT29-MTX co-cultures, which simulates the human intestinal mucosa, against the detrimental effect of ETEC. This effect was achieved with all MV preparations, however MV from strain BG-R46 gave significantly better protection than MV from DSM 17938 (Figure 5B). Although the mechanism of protection is currently unknown the MV carry two types of molecules, LTA and 5′ nucleotidase (5′NT), with a potential link to epithelial protection. LTA detected on the surface of the MV (Figure 4) has in several studies been described to protect the epithelial barrier *via* interactions with TLR2 (Gu et al., 2016). 5′NT was detected both in the proteome analysis (Supplementary Table S1) and by measuring the enzyme activity (Table 2). This extracellular protein converts AMP to adenosine, which is a potent signal molecule that among other functions has a role in strengthening tissue barriers *via* interacting with the A2a and A2b receptors (Madara et al., 1993; Strohmeier et al., 1997; Lennon et al., 1998). Interestingly, at a multiplicity of MV per epithelial cell (MOM) of 10, MV from *L. reuteri* BG-R46, which has the highest 5′NT activity, showed the strongest effect on epithelial cell integrity (Figure 5B). However, this was not supported by the proteomic analysis. A possible explanation for this discrepancy could be that the 5′NT is exposed differently on the two strains and steric hindrance thereby prevent the trypsin cleavage used in the surface shaving protocol. It should be highlighted that the proteomics analysis included in this study was not performed for quantitative measures. We further investigated how MV interacts with intestinal epithelial cells, and with confocal microscopy we demonstrated that MV were taken up by the cells. Thus, MV could potentially interact with apical, intracellular or basolateral targets of epithelial cells. These interactions may also mediate transportation of vesicles *in vivo* to distant parts of the body. A recent review (Chronopoulos and Kalluri, 2020) discuss bacterial membrane vesicle presence in the blood as well as their dissemination throughout the body and tentative access to the brain. Interestingly, adenosine receptor signaling has been described as an access route through the blood brain barrier (Bynoe et al., 2015). Further studies addressing systemic MV effects is warranted.

The immunostimulatory activity of lactobacilli is both species and strain dependent. MV from both *L. reuteri* DSM 17938 and *L. reuteri* BG-R46 showed immunostimulatory activity by inducing secretion of IL-6 and IL-1β in PBMC (Figure 7), two cytokines described as markers for immune maturation in infants (Rabe et al., 2020). Interestingly, MV from both strains also blocked the secretion of IFN-γ and reduced secretion of TNF-α induced by *S. aureus.* Thus, potentially *L. reuteri* derived MV can modulate the immune system, both by stimulating basal immune responses and dampen pathogen-induced inflammation. MV isolated from LGG stimulated IL-6 and IL-1β but did not reduce IFN-γ and TNF-α, suggesting that MV from different lactobacilli interact with immune cells through different mechanisms. As discussed above, the MV from *L. reuteri* carry LTA. This molecule is one of the major surface components of Gram-positive bacteria and their vesicles known to be involved in immunomodulation (Mizuno et al., 2020; Champagne-Jorgensen et al., 2021) as well as in attachment to host cells (Beachey and Courtney, 1987; Ohshima et al., 1990). Wang et al. (Wang et al., 2000) have reported that LTA from *S. aureus* induce IL-6 production in both T cells and monocytes in a human whole blood model. An interesting link to epithelial protection is that IL-6 has been described to protect the mucosal barrier by upregulating the expression of keratin-8 and keratin-18 (Wang et al., 2007), which could be an additional mechanism for how the MV enhance the epithelial barrier integrity.

The above discussed adenosine is also an important modulator of inflammation and its anti-inflammatory effects have been well established in different models (Colgan and Eltzschig, 2012; Bowser et al., 2017; Liu et al., 2018). Extracellular adenosine reduces expression of pro-inflammatory cytokines, such as TNF-α in IECs (Alam et al., 2009). This, together with the fact that MV from LGG, lacking 5′NT activity, did not dampen TNF-α, suggests that the 5’NT contributes to the observed anti-inflammatory effect. This is supported by previously observed contrasting effects of *L. reuteri* DSM 17938 and LGG in a T-reg deficient mice model where only *L. reuteri* increased the abundance of adenosine metabolites, such as inosine, in plasma (Liu et al., 2021). This altogether indicates that 5′NT located on the surface of MV, similar to CD73 on T-reg cells (Alam et al., 2009), play an important role in the regulation of mucosal immune responses. Intriguingly, CD8 T-cells have been shown to secrete CD73 positive vesicles that contribute to battling inflammation in inflamed tissues (Schneider et al., 2021).

We also detected a dextran sucrase, having the highest #PSM and #peptide values, on the surface of MV. This enzyme catalyze glycosidic bindings in sucrose and utilize the glucose molecules to synthesize extracellular polysaccharides (EPS) (Monchois et al., 1999). It has previously been described that *L. reuteri* DSM 17938 express the same dextran sucrase that was found on the MV, and that the produced EPS can inhibit ETEC from adhering to cultivated epithelial cells and reduce the proinflammatory effects of the pathogen (Ksonzekova et al., 2016). It is intriguing that also the MV have the capacity to produce EPS, and in that way add a component to the arsenal of bioactivities.

*Limosilactobacillus reuteri* DSM 17938 has proven to be effective for treatment of infantile colic (Urbanska and Szajewska, 2014). The etiology of colic is not fully understood, but it is plausible that visceral pain is one of the main reasons for the extensive crying in colicky infants (Geertsma and Hyams, 1989). In mouse models, DSM 17938 has previously been shown to antagonize one of the main pain receptors, namely TRPV1 (Perez-Burgos et al., 2015), and by that decrease firing of pain signals from the intestine. Here, we demonstrate that also the MV from DSM 17938 and BG-R46 are able to antagonize TRPV1, perhaps to an even greater extent than *L. reuteri* DSM 17938 cells, which was reported by Perez-Burgos et al. (2015). The authors reported a decreased TRPV1 ionic current in a similar dorsal root ganglion (DRG) model, evoked by 10^9^ CFU/ml DSM 17938, with a reduction value of 42%. Here we report a reduction value of 75% evoked by the same concentration of MV from the same strain, indicating a higher antagonistic effect of MV than from bacterial cells. The higher antagonistic effect from MV could relate to their advantageous size that could help them access the target more easily or being internalized as shown in Figure 6. Adenosine which is a result from the above-described 5′NT activity, could play an important role in TRPV1 antagonism. This effector molecule has previously been shown to antagonize the TRPV1 receptor (Puntambekar et al., 2004) but future studies are needed to clarify if this is the mechanism by which *L. reuteri* interacts with the receptor.

The etiology of infantile colic is not fully understood (Daelemans et al., 2018). However, the models used in this paper are all well connected to the hypothesis around the amelioration of infantile colic by DSM 17938 and covers mechanisms related to potential underlying disturbances (Supplementary Figure S3). Of course, those *in vitro* models are far from an infant gastrointestinal tract and whether the MV has the same functions *in vivo* remains to be elucidated. We can however conclude that *L. reuteri* MV reproduce the mechanistic actions by which strain DSM 17938 is thought to ameliorate infantile colic. Additionally, MV from strain BG-R46 have comparable and in some biological models stronger activities than MV from DSM 17938, suggesting that BG-R46 would be an interesting candidate for further evaluation in clinical trials addressing infantile colic. While MV exposed protein profiles from the two strains were highly similar, the 5′NT activity was significantly higher in BG-R46, and we hypothesize that adenosine could be an important mediator of probiotic effects. To the best of our knowledge, MV carrying the host-interaction enzymes 5′NT and glucansucrase has never been reported before. Finally, the ability of membrane vesicles (MV) from *L. reuteri* to protect epithelial cells from detrimental effects of ETEC, modulate cytokine responses and antagonize activation of TRPV1, altogether demonstrates a novel type of multifunctionality of MV from a mutualistic bacterium. These findings could contribute to the development of new innovative and more efficient probiotic or postbiotic products (Wegh et al., 2019).

## Data availability statement

The datasets presented in this study can be found in online repositories. The names of the repository/repositories and accession number(s) can be found in the article/Supplementary material. https://osf.io/evuy2/?view_only=2946487314cb43039520855a0dd6c4e8

## Ethics statement

The animal study was reviewed and approved by ethical permit no. 76–2013 and Regional Ethics Committee at the Karolinska Institute, Stockholm, Sweden Dnr 2014/2052–32, experiments were performed in accordance with European and Swedish animal welfare regulations.

## Author contributions

YP and LEL: conceptualization, data curation, formal analysis, investigation, methodology, project administration, validation, visualization and writing-initial draft, review and editing. MMF and DA: data curation and formal analysis. AP: methodology. HB and ES: funding acquisition, supervision. RK: data curation, investigation, resources, visualization. HJ: conceptualization, resources, supervision. SR: conceptualization, data curation, funding acquisition, project administration, resources, supervision, validation, writing-review and editing. All authors contributed to the article and approved the submitted version.

## Funding

This work was supported by BioGaia AB (Stockholm, Sweden); The Swedish Research Council (2020-01839); The Swedish Cancer Foundation (20 1117 PjF 01 H); Swedish University of Agricultural Sciences (SLU.ua 2016.1.1-2756; SLU.ua.2017.1.1.1-2416) and LivsID (food science-related industry PhD program), financed by the Swedish Government (governmental decision N2017/03895).

## Conflict of interest

SR, LEL, and HB are all employees of BioGaia AB.

The remaining authors declare that the research was conducted in the absence of any commercial or financial relationships that could be construed as a potential conflict of interest.

## Publisher’s note

All claims expressed in this article are solely those of the authors and do not necessarily represent those of their affiliated organizations, or those of the publisher, the editors and the reviewers. Any product that may be evaluated in this article, or claim that may be made by its manufacturer, is not guaranteed or endorsed by the publisher.

## Footnotes

^1^ https://www.uniprot.org

^2^ http://www.moonlightingproteins.org

^3^ https://www.ncbi.nlm.nih.gov/protein/

^4^ http://www.moonlightingproteins.org
