# Supplementary material for: Extracellular membrane vesicles from Limosilactobacillus reuteri strengthen the intestinal epithelial integrity, modulate cytokine responses and antagonize activation of TRPV1
Source: Front Microbiol. 2022 Nov 17;13:1032202. doi: 10.3389/fmicb.2022.1032202 (PMC9712456; doi:10.3389/fmicb.2022.1032202)
Supplement: Supplementary file 1 [file Data_Sheet_1.docx]

Supplementary Material

#
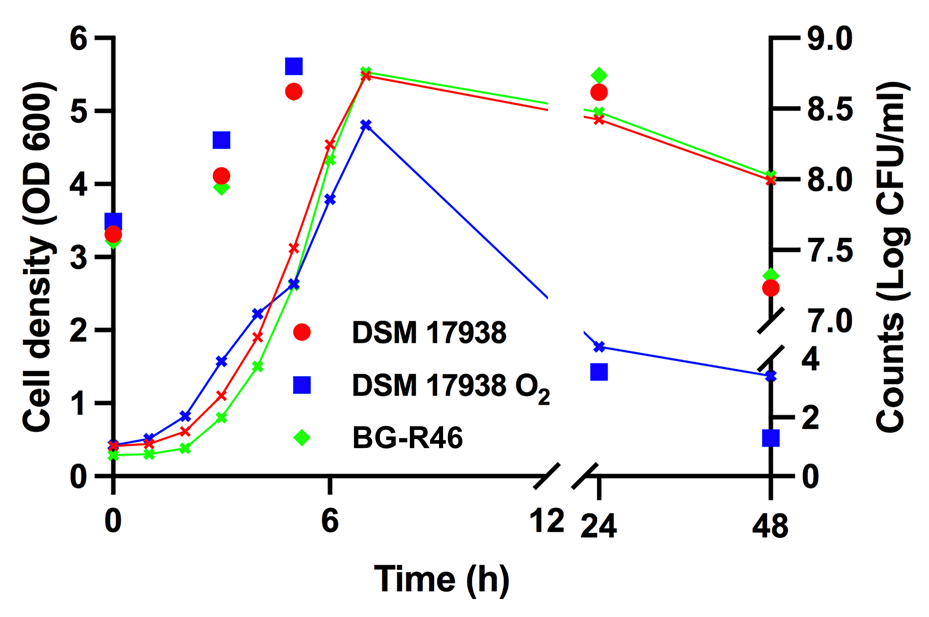


# Figure 1. Growth curve of *L. reuteri* DSM 17938 cultivated in flasks with/without agitation (O_2_ stress) and BG-R46 cultivated without agitation. The optical densities (crosses) were measured by using a spectrophotometer and counts of live bacteria (large symbols) were counted by plating on MRS agar plates. color key: red = DSM 17938, blue = DSM 17938 O_2_, green = BG-R46.

#
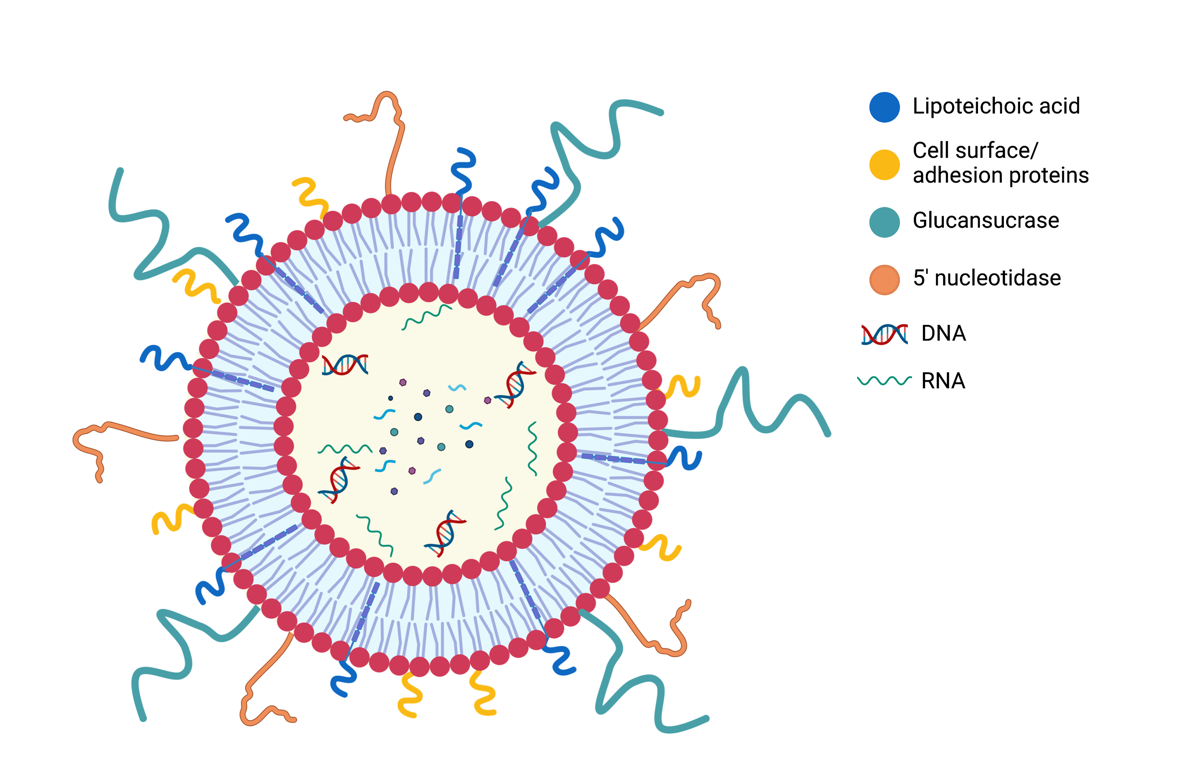
 Figure 2. Schematic overview of bioactive molecules present in MV of *L. reuteri.* Color and structure code is denoted in the figure.

#
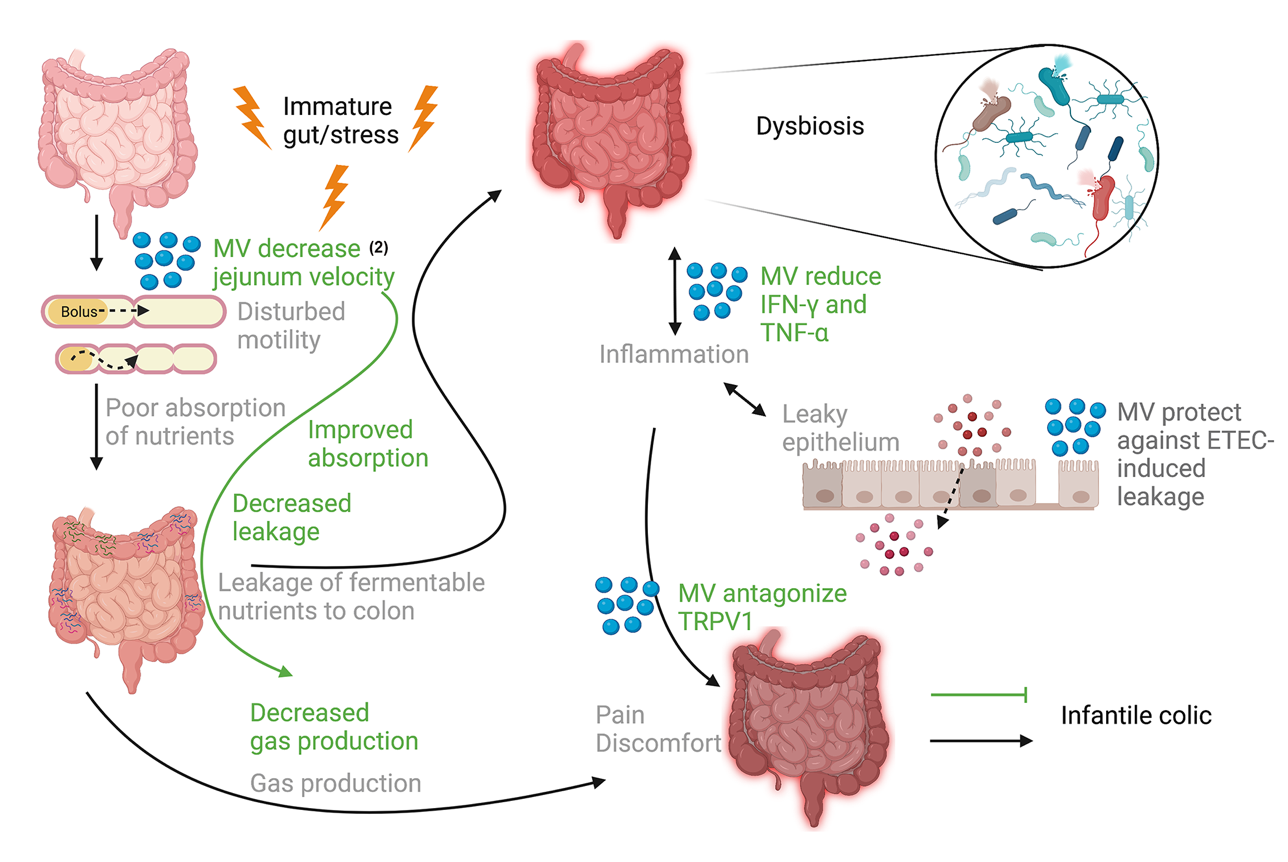


# Figure 3. Schematic overview of how *L. reuteri* MV reproduce the proposed mechanism by which *L. reuteri* ameliorate infantile colic. Green text and markers indicate functions of MV while gray and black indicate the colic phenotype. (2) West, C.L., et al., Microvesicles from Lactobacillus reuteri (DSM-17938) completely reproduce modulation of gut motility by bacteria in mice*.* PLoS One, 2020. 15(1): p. e0225481.

Table 1. A selection of the most abundant proteins detected on MV derived from *L. reuteri* strains DSM 17938 and BG-R46 harvested at 24 h. After cultivation of the bacteria in MRS broth the MV were isolated by ultracentrifugation. The proteome of the MV preparations was determined by LC-MS/MS analysis of peptides released from the MV by trypsin digestion. Both the number of unique peptide sequences (# peptides) and the total number of peptide to spectrum matches (#PSM) are displayed. Triplicate analysis were performed.

| Annotation | | Accession ID^$^ | Domains; additional | Mw | Predicted | MV * | | | |
| --- | --- | --- | --- | --- | --- | --- | --- | --- | --- |
|  | |  | **annotation** | (kDa) | **localization^€^** | **DSM 17938**  # peptides # PSM | | **BG-R46**  # Peptides # PSM | |
| Adhesion and cell interactions | | |  |  |  |  |  |  |  |
| 5’-nucleotidase | HMPREF0538_20056; Lr1025 | | LPXTG anchor; 5'-nucleotidase | 80.7 | Cell surface, LPxTG | 25±1 | 54±10 | 17±5 | 23±5 |
| LPXTG cell wall anchor domain-containing protein | HMPREF0538_20063; Lr1487 | | LPXTG anchor; 4x Coiled coil; putative type IV pilus biogenesis protein PilP; Rib/alpha-like repeat | 68.3 | Cell surface, LPxTG | 17±2 | 19±3 | 8±1 | 8±1 |
| MucBP protein | HMPREF0538_20356; Lr1612 | | KxYKxGKxW signal peptide; 5x MucBP motifs | 172.3 | Cell surface, GW | 32±6 | 55±4 | 14±3 | 16±2 |
| YSIRK-type signal peptide-containing protein | HMPREF0538_20775 HMPREF0538_20774; Lr1694 | | YSIRK signal; 9x Rib_alpha; putative adhesion protein; LPXTG anchor | 187.8 | Cell surface, LPxTG | 21±1 | 38±2 | 22±2 | 34±3 |
|  | | |  |  |  |  |  |  |  |
| Moonlighting proteins | | |  |  |  |  |  |  |  |
| Elongation factor G | HMPREF0538_20596 | | Adhesin, binds salivary mucin MUC7 | 76.7 | Cytoplasm | 24±3 | 37±5 | 21±1 | 28±1 |
| Phosphoketolase | HMPREF0538_20863 | | Mucin binding | 91.4 | Cytoplasm | 30±6 | 64±1 | 25±2 | 39±2 |
| 6-Phosphogluconate dehydrogenase | HMPREF0538_20952 | | Adhesin | 53.4 | Cytoplasm | 26±5 | 86±6 | 27±2 | 86±5 |
| Aminopeptidase PepN | HMPREF0538_21097 | | Surface located peptidase^1^ | 95.3 | Cytoplasm | 35±5 | 112±26 | 26±6 | 42±8 |
| ABC transporter, substrate-binding protein | HMPREF0538_21501; Lr0793 | | Collagen/Mucus binding protein CnBP ^2^ | 28.5 | Cell surface, pI >9 | 27±3 | 91±32 | 21±3 | 46±6 |
| 60 kDa chaperonin | HMPREF0538_21561 | | GroEL; binds mucins, epithelial cells and invertase | 57.1 | Cytoplasm | 33±3 | 76±14 | 22±2 | 33±3 |
| Glyceraldehyde-3-phosphate dehydrogenase | HMPREF0538_21606 | | Mucus- and cell-binding protein | 37.0 | Cytoplasm | 20±1 | 286±129 | 19±2 | 166±30 |
| Phosphoglycerate kinase | HMPREF0538_21607 | | Mucin binding | 43.0 | Cytoplasm | 33±4 | 97±25 | 26±1 | 57±5 |
| Enolase | HMPREF0538_21609 | | Fibronectin/plasminogen/ laminin binding | 49.9 | Cytoplasm | 19±1 | 96±28 | 19±2 | 58±5 |
| Pyruvate kinase | HMPREF0538_22005 | | Invertase binding | 51.8 | Cytoplasm | 25±2 | 73±16 | 24±2 | 49±3 |
| Glucose 6-phosphate isomerase | HMPREF0538_21641 | | Laminin and collagen binding | 50.4 | Cytoplasm | 21±3 | 80±21 | 20±2 | 38±4 |
|  | | |  |  |  |  |  |  |  |
| EPS production | | |  |  |  |  |  |  |  |
| Peptidase, M23 family | | HMPREF0538_20383; Lr0899 | 3x Cell wall-binding repeats (CW); Peptidase_M23; carbohydrate transport | 101.1 | Cell surface, GW | 30±2 | 94±32 | 21±1 | 34±3 |
| Dextran sucrase | | HMPREF0538_20764; Lr1943 | KxYKxGKxW signal peptide; glycosyl hydrolase family 70; 5x Cell wall-binding repeats (CW); | 200.3 | Cell surface, GW | 97±9 | 259±61 | 59±11 | 79±13 |
|  | |  |  |  |  |  |  |  |  |
| Cell wall modulation | |  |  |  |  |  |  |  |  |
| Penicillin-binding protein Pbp2b, transpeptidase | | HMPREF0538_20221; Lr0889 | Transpeptidase (cell wall biosynthesis) | 76.0 | Membrane, cell surface | 30±1 | 101±46 | 28±1 | 54±4 |
| N-acetylmuramoyl-L-alanine amidase | | HMPREF0538_20363; Lr1039 | KxYKxGKxW signal peptide; Amidase domain | 96.5 | Cell surface, GW | 24±4 | 34±3 | 9±3 | 10±3 |
| Peptidase | | HMPREF0538_20382; Lr0898 | KxYKxGKxW signal peptide; Peptidase_C39-like | 93.2 | Cell surface, GW | 22±4 | 34±1 | 8±2 | 9±3 |
| Transferase | | HMPREF0538_21056; Lr1829 | Cell wall biogenesis | 51.4 | Membrane, cell surface | 24±2 | 46±10 | 19±2 | 29±2 |
| Peptidoglycan hydrolase | | HMPREF0538_21064; Lr1822 | KxYKxGKxW signal peptide; Glucosaminidase; 4x LysM; | 60.5 | Cell surface, LysM | 29±3 | 164±64 | 30±1 | 134±33 |
| D-alanyl-lipoteichoic acid biosynthesis protein DltD | | HMPREF0538_21428; Lr1649 | D-alanyl-lipoteichoic acid biosynthesis protein | 49.4 | Cell surface, Lipoprotein | 20±4 | 58±7 | 17±2 | 39±1 |
| DD-transpeptidase, Pbp1a | | HMPREF0538_22189; Lr0545 | Transpeptidase (cell wall biosynthesis) | 81.9 | Membrane, cell surface | 32±4 | 135±40 | 28±3 | 52±5 |
|  | |  |  |  |  |  |  |  |  |
| Other membrane proteins | | |  |  |  |  |  |  |  |
| Cation transport ATPase; MraZ | | HMPREF0538_20821 | Cation ATPase | 100.1 | Membrane | 36±4 | 84±12 | 35±1 | 67±6 |
| ABC transporter, ATP-binding protein | | HMPREF0538_21067 |  | 71.8 | Membrane | 27±2 | 62±7 | 23±1 | 45±11 |
| Uncharacterized surface protein | | HMPREF0538_21191; Lr1863 | C-terminal membrane anchor; 3x Coiled coil | 103.6 | Membrane, cell surface | 26±2 | 36±2 | 29±1 | 48±7 |
| Protein translocase subunit SecA | | HMPREF0538_21575 |  | 90.4 | Membrane | 27±6 | 37±5 | 23±2 | 34±4 |
| ATP synthase subunit alpha | | HMPREF0538_21685 | Proton-transporting ATP synthase activity | 55.2 | Membrane | 30±1 | 189±68 | 30±2 | 176±29 |
|  | |  |  |  |  |  |  |  |  |

$ Uniprot identity, https://www.uniprot.org/; Identity in Båth et al., 2005. Moonlighting protein identity, <http://www.moonlightingproteins.org>.

€ Predicted by analysis with SignalP and TMHMM; Cell surface localization according to Båth et al., 2005. Raw data file sorted after mean # of peptides in DSM 17938 MV. (1) Marquart, M.E., Pathogenicity and virulence of Streptococcus pneumoniae: Cutting to the chase on proteases. Virulence, 2021. **12**(1): p. 766-787.

* Average number of hits ± S.D.
